# Sol-Gel Derived Adsorbents with Enzymatic and Complexonate Functions for Complex Water Remediation

**DOI:** 10.3390/nano7100298

**Published:** 2017-09-28

**Authors:** Roman P. Pogorilyi, Ievgen Pylypchuk, Inna V. Melnyk, Yurii L. Zub, Gulaim A. Seisenbaeva, Vadim G. Kessler

**Affiliations:** 1 Chuiko Institute of Surface Chemistry of National Academy of Sciences of Ukraine, 17, General Naumov Street, 03164 Kyiv, Ukraine; pogorilyi_r@ukr.net (R.P.P.); ievgen.pylypchuk@slu.se (I.P.); in.melnyk@gmail.com (I.V.M.); yurii.zub@gmail.com (Y.L.Z.); 2 Department of Molecular Sciences, Swedish University of Agricultural Sciences, 750 07 Uppsala, Sweden

**Keywords:** magnetite, urease, DTPA, heavy metal adsorption, urea decomposition, copper, cadmium

## Abstract

Sol-gel technology is a versatile tool for preparation of complex silica-based materials with targeting functions for use as adsorbents in water purification. Most efficient removal of organic pollutants is achieved by using enzymatic reagents grafted on nano-carriers. However, enzymes are easily deactivated in the presence of heavy metal cations. In this work, we avoided inactivation of immobilized urease by Cu (II) and Cd (II) ions using magnetic nanoparticles provided with additional complexonate (diethylene triamine pentaacetic acid or DTPA) functions. Obtained nanomaterials were characterized by Fourier transform infrared spectroscopy (FTIR), thermogravimetric analysis (TGA), and scanning electron microscopy (SEM). According to TGA, the obtained Fe_3_O_4_/SiO_2_-NH_2_-DTPA nanoadsorbents contained up to 0.401 mmol/g of DTPA groups. In the concentration range *C*_eq_ = 0–50 mmol/L, maximum adsorption capacities towards Cu (II) and Cd (II) ions were 1.1 mmol/g and 1.7 mmol/g, respectively. Langmuir adsorption model fits experimental data in concentration range *C*_eq_ = 0–10 mmol/L. The adsorption mechanisms have been evaluated for both of cations. Crosslinking of 5 wt % of immobilized urease with glutaraldehyde prevented the loss of the enzyme in repeated use of the adsorbent and improved the stability of the enzymatic function leading to unchanged activity in at least 18 cycles. Crosslinking of 10 wt % urease on the surface of the particles allowed a decrease in urea concentration in 20 mmol/L model solutions to 2 mmol/L in up to 10 consequent decomposition cycles. Due to the presence of DTPA groups, Cu^2+^ ions in concentration 1 µmol/L did not significantly affect the urease activity. Obtained magnetic Fe_3_O_4_/SiO_2_-NH_2_-DTPA-Urease nanocomposite sorbents revealed a high potential for urease decomposition, even in presence of heavy metal ions.

## 1. Introduction

Development of novel multifunctional nanoadsorbents for water remediation, both from organic and inorganic components, is a very important task for modern science and technology [1,2,3,4,5]. Recently, a number of adsorbents that are effective against some toxic metals and organic pollutants have been proposed [6,7,8,9,10,11,12,13,14,15,16]. Some of them are magnetic, and related to “green”, eco-friendly nanomaterials [7,8,9,13,14,15,16]. Unfortunately, no effective “green” nanomaterials have been reported so far for simultaneous removal of both organic and inorganic pollutants from water solutions. 

The removal of urea from wastewaters is an important problem in numerous areas, such as urea-producing industries, agriculture and forestry, food production, and medicine [1,17]. In the environment, the sources of urea are from the industries that utilize it, as well as from release from arable soils as fertilizer wastewater effluents, and also in effluents from households, but primarily from urine excretion by animals [1]. Before its discharge into the environment, the wastes need to have their urea content reduced to less than 0.006% [17]. Removal of urea from aqueous solution by adsorption is thus a highly addressed topic [17,18]. The adsorption capacity of amino-functionalized SBA-16 mesoporous silica derived adsorbents was shown to be able to reach up to 500 mg/g, but their regeneration was an apparent problem [18]. It should also to be mentioned that ordered mesoporous silicas remain rather expensive materials, which potentially renders their broad scale application in water purification.

One of the most efficient ways to remove urea from water solutions is by using urease enzyme [19,20]. Ureases are metalloenzymes from the hydrolase class that catalyze the hydrolysis of urea into CO_2_ and NH_3_ [21,22,23]. They can be adsorbed on silica-based matrices with relatively high capacity [24], but can easily be washed out if not chemically bound to them [19,20,25].

The main problem in the application of urease is its inactivation in the presence of some heavy metal ions [25]. Our idea was to avoid urease inactivation by heavy metal ions using, on one hand, its stabilization by immobilization on magnetic nanoparticles and, on the other hand, providing these particles with strong metal-chelating function, e.g., a derivative of diethylenetriaminepentaacetic acid (DTPA). In this work we focused on the development of “green” multifunctional magnetic nanomaterial, a perspective for urea decomposition and the effective removal of some of the harmful heavy metals from water solutions.

## 2. Results

**Scheme 1:**
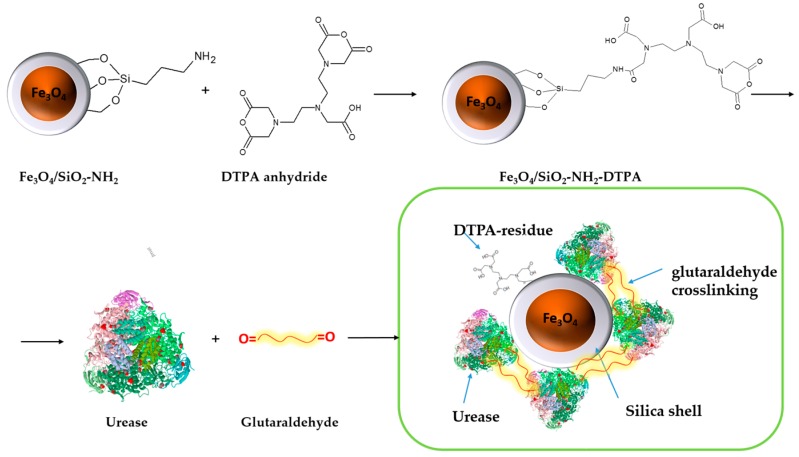
Urease immobilization on the surface of the Fe_3_O_4_/SiO_2_-NH_2_- diethylene triamine pentaacetic acid (DTPA) nanocomposite.

The process of urease immobilization, as well as the Scheme 1 for the synthesis of the Fe_3_O_4_/SiO_2_-NH_2_-DTPA nanocomposite, is presented below. Briefly, magnetite particles obtained by controlled co-precipitation [26] were coated by a silica shell using sol-gel condensation in basic medium (modified Stoeber method) in order to protect them against oxidation and the risk of dissolution in acidic media. The core-shell particles were then grafted with organic aminopropyl functions by condensation with aminopropyltriethoxysilane. The particles applied in this work were produced following slightly modified versions of the recently developed procedures employing NH_4_F as catalyst [19].

Obtained aminosilica shells reacted with DTPA anhydride, yielding a DTPA-containing coating with up to 0.401 mmol DTPA per g adsorbent. Urease adsorbed and crosslinked on these shells revealed, as demonstrated below, increased stability and activity in the presence of heavy metal ions—Cu (II) and Cd (II) in acidic media.

### 2.1. Adsorbent Characterization

#### 2.1.1. Scanning Electron Microscopy

**Figure 1:**
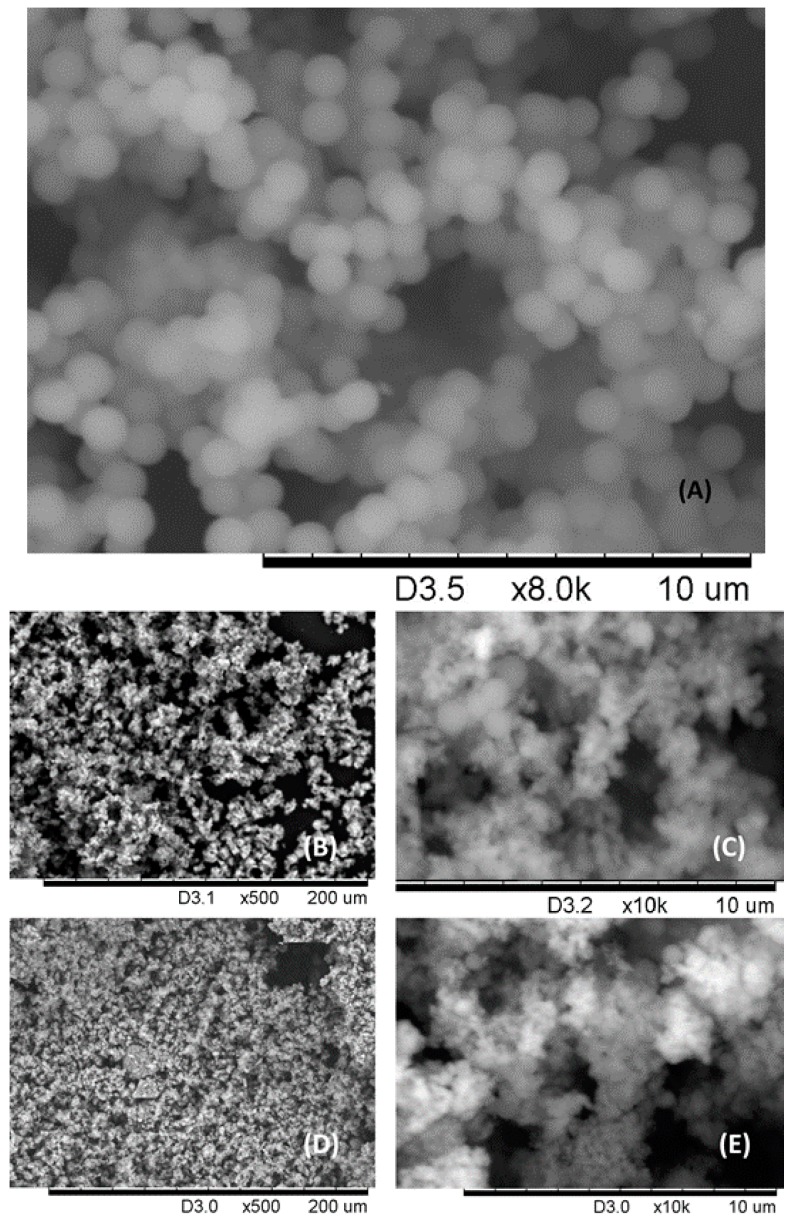
Scanning electron microscope (SEM) images of the: (**A**) Fe_3_O_4_/SiO_2_ spherical aggregates of nanoparticles; (**B**,**C**) Fe_3_O_4_/SiO_2_-NH_2_ nanocomposites; (**D**,**E**) Fe_3_O_4_/SiO_2_-NH_2_-DTPA nanocomposites under different magnifications.

SEM images of the: Fe_3_O_4_/SiO_2_, Fe_3_O_4_/SiO_2_-NH_2_ and Fe_3_O_4_/SiO_2_-NH_2_-DTPA nanocomposites were recorded in order to investigate the shape and surface morphology of the obtained nanocomposites. In the SEM images (Figure 1A–E), the nanoparticles were observed to have a close to spherical shape, forming coalesced spherical aggregates up to 800 nm in size. On surface modification, the aggregates were split into smaller blocks because of the higher surface charge of the modified individual particles (please, see the discussion below in Section 2.1.4). 

#### 2.1.2. Thermogravimetric Analysis

For the Fe_3_O_4_/SiO_2_-NH_2_ composite (Appendix B, Figure A2a), the low-temperature mass loss from 30 to 170 °C corresponds to evaporation of the adsorbed water (6.664%). In the temperature range 200–350 °C mass losses for the Fe_3_O_4_/SiO_2_-NH_2_ composite reach about 9.87%. Decomposition at that temperature range was most likely associated with the destruction of the organic component on the surface, in particular by the ≡ Si–(CH_2_)_3_–NH_2_ groups’ decomposition.

In the case of the Fe_3_O_4_/SiO_2_-NH_2_-DTPA nanocomposite (Appendix B, Figure A2b), after evaporation of water (5.316%), mass loss in the temperature range 200–350 °C was 17.383%. The observed increase in the mass of the organic component in the thermogram clearly resulted from DTPA grafting on the Fe_3_O_4_/SiO_2_-NH_2_ composite surface.

The molar weight of the aminopropyl radical (–(CH_2_)_3_–NH_2_) was 58 g/mol. This permitted the conversion of the mass loss into the content of amino groups on the surface, as it was 1.69 mmol/g of the Fe_3_O_4_/SiO_2_–NH_2_ composite. In the case where one molecule of DTPA anhydride reacted with only one NH_2_ group of the aminopropyl radical, the theoretical maximum of DTPA grafting should be: 357.32 mg/mmol × 1.69 mmol/g = 603.87 mg/g. For the Fe_3_O_4_/SiO_2_-NH_2_-DTPA nanocomposite the weight loss was 17.83%, which corresponded to 0.401 mmol/g of –(CH_2_)_3_-NH_2_-DTPA residue (433.43 mg/mmol).

#### 2.1.3. Fourier Transform Infrared Spectroscopy

**Figure 2:**
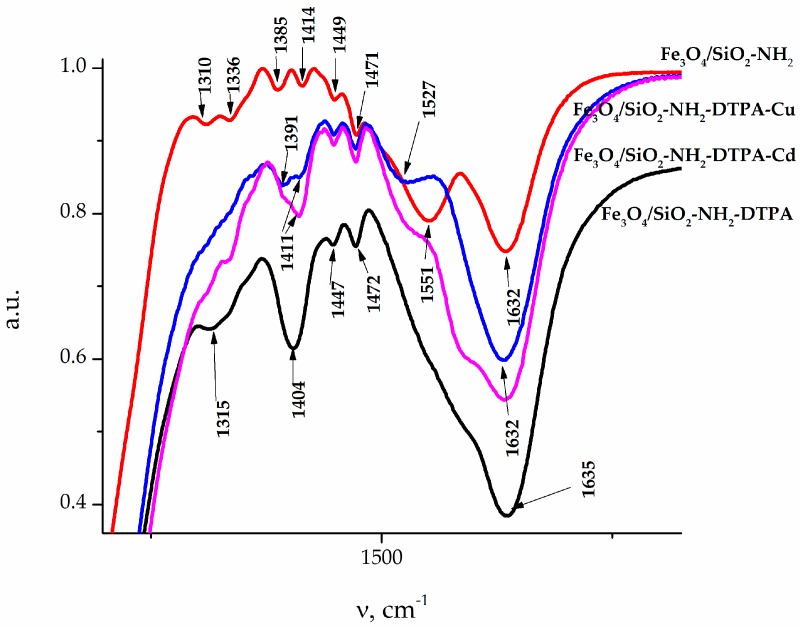
Fourier transform infrared spectroscopy (FTIR) spectra of the Fe_3_O_4_/SiO_2_-NH_2_ (red), Fe_3_O_4_/SiO_2_-NH_2_-DTPA (black), and Fe_3_O_4_/SiO_2_-NH_2_-DTPA nanocomposites, bearing Cu^2+^ (blue) and Cd^2+^ (purple).

**Figure A1:**
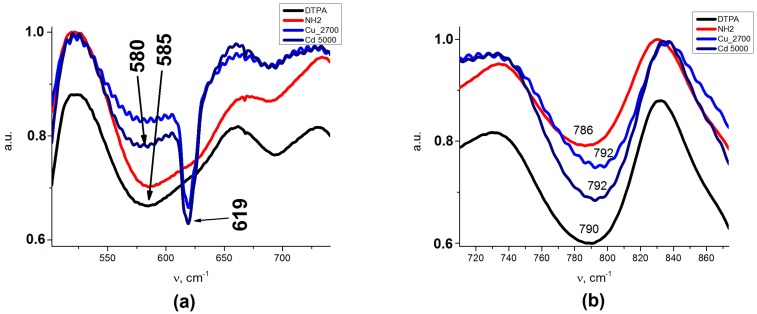

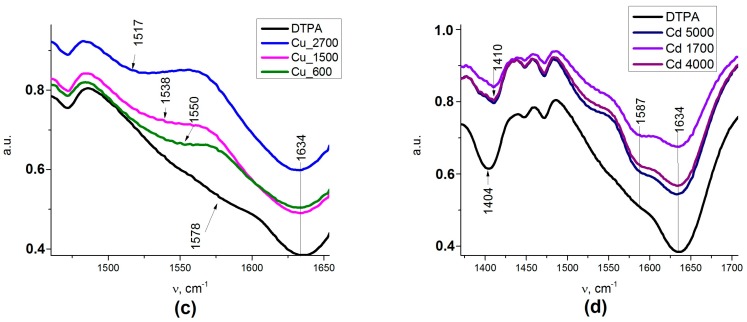
FTIR of the Fe_3_O_4_/ SiO_2_-NH_2_- DTPA-Cu/Cd nanocomposites: (**a**) 500-700 cm^−1^ (Me–O bond vibrations region); (**b**) 740-820 cm^−1^ (C–C bond vibrations region); (**c**) 1500-1650 cm^−1^ (ν C=O vibrations region); (**d**) 1400-1700 (COOH vibrations region).

**Figure A2:**
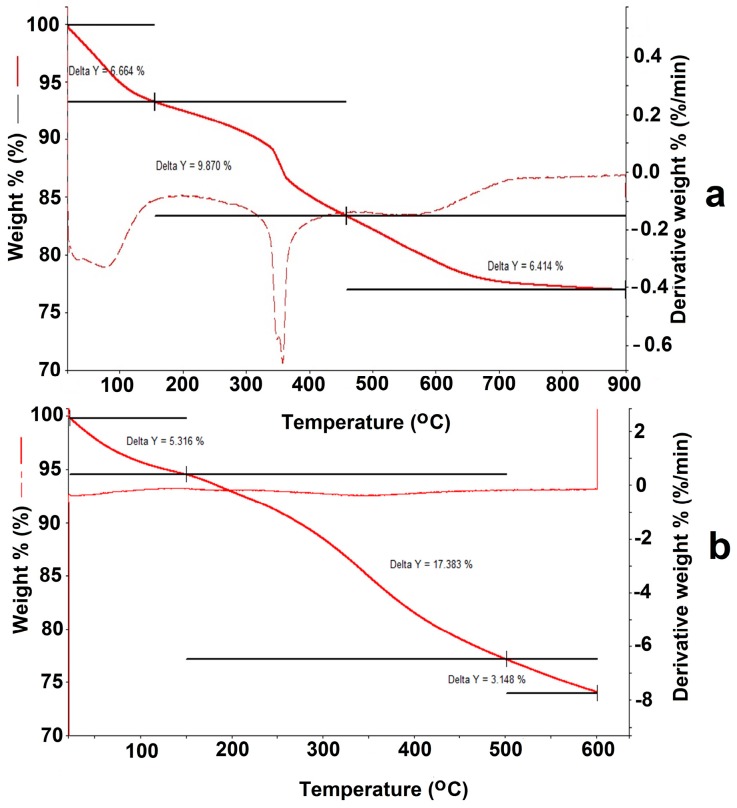
Thermograms of Fe_3_O_4_/SiO_2_-NH_2_ (**a**) and Fe_3_O_4_/SiO_2_-NH_2_-DTPA (**b**) nanocomposites.

The IR spectra of the samples (Figure 2) also revealed absorption bands at 456 cm^−1^, which could be attributed to iron oxide. The adsorption bands (AB) at 1154–1050 and 787 cm^−1^ belonged to the symmetrical and anti-symmetric vibrations of ν(Si–O–Si). The band of medium intensity at 1541 cm^−1^ in the IR spectra of the sample referred to the bending vibration δ(NH_2_) of the amino group [27]. A low intensity AB at 2974–2935 cm^−1^, 1473–1445 cm^−1^ and 1333–1329 cm^−1^ could be assigned to alkyl stretching mode vibrations ν(C–H) of the ethylene bridge in DTPA and the propyl chain of the aminopropyl triethoxy silane (APTES) linkers. Thus, the synthesized samples featured a magnetite core coated with a polysiloxane shell, bearing a functional aminopropyl, as well as DTPA groups on the surface [28,29]. The AB at 1401 cm^−1^ could be associated with asymmetric (ν_as_) stretching vibrations of the –COO^−^ group of DTPA. A more detailed presentation of FTIR results can be found in Appendix A (Figure A1a–d, Table A1 and Table A2).

#### 2.1.4. Point of Zero Charge 

**Figure 3:**
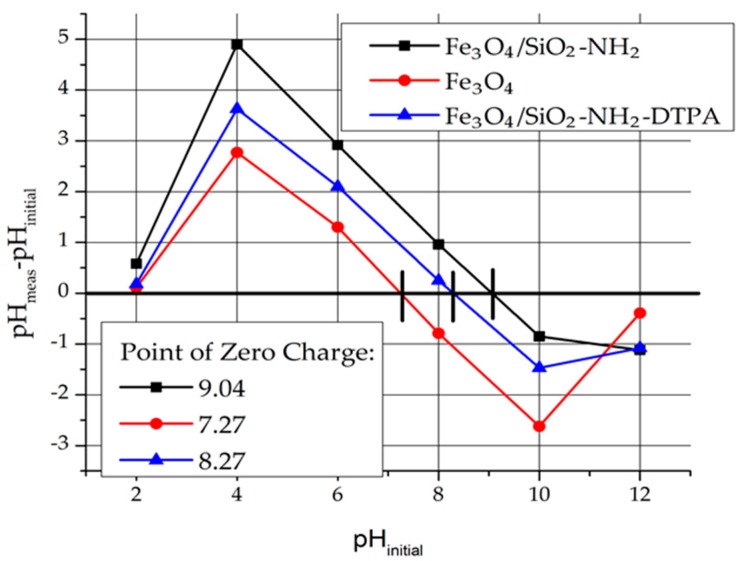
Surface charge of the core-shell particles of Fe_3_O_4_/SiO_2_-NH_2_ and Fe_3_O_4_/SiO_2_-NH_2_-DTPA nanocomposites determined according to the pH drift method [31,32].

In order to determine the charge of the adsorbent surface, the Point of Zero Charge (Pzc) was determined (Figure 3). For instance, pH_pzc_ for Fe_3_O_4_ is 7.25. That means that at a pH below Pzc, the magnetite surface is charged positively. The surface charge of Fe_3_O_4_ in aqueous medium is due to the presence of Fe-OH groups (≡ FeOH + H^+^ → FeOH_2_^+^).

For the Fe_3_O_4_/SiO_2_-NH_2_ and Fe_3_O_4_/SiO_2_-NH_2_-DTPA nanocomposites, pH_pzc_ = 9.04 and 8.27 respectively were determined.

Due to the presence of NH_2_ groups, the surface of Fe_3_O_4_/SiO_2_-NH_2_ composite was more basic (Fe_3_O_4_/SiO_2_-NH_2_ + H^+^ → Fe_3_O_4_/SiO_2_-NH_3_^+^) and shifted to higher values of Pzc. The surface charge of Fe_3_O_4_/SiO_2_-NH_2_-DTPA decreased because of amino group deprotonation by DTPA residues (Fe_3_O_4_/SiO_2_-NH_2_-DTPA^4−^ + Fe_3_O_4_/SiO_2_-NH_3_^+^ → Fe_3_O_4_/SiO_2_-NH_2_-DTPA^3−^ + Fe_3_O_4_/SiO_2_-NH_2_) [30,31,32]. 

### 2.2. Cu (II) and Cd (II) Adsorption

**Figure 4:**
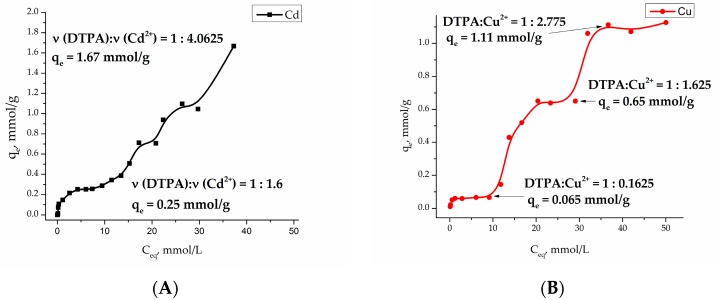
Adsorption isotherms of Cd (II) (**A**) and Cu (II) (**B**) on Fe_3_O_4_/SiO_2_-NH_2_-DTPA nanocomposite.

Investigation of the adsorption of heavy metal cations Cu (II) and Cd (II) at pH 4.91 (acetate buffer) on the Fe_3_O_4_/SiO_2_-NH_2_-DTPA core-shell particles became a multistep process with a several plateaus, indicating the formation of a number of different surface complexes (see Figure 4).

Adsorption isotherms were analyzed by fitting them to Langmuir [33] and Freundlich [34] isotherm models. The Langmuir model is based on the assumption that maximum adsorption occurs when a saturated monolayer of solute molecules is present on the adsorbent surface, and the energy of adsorption is constant with no migration of adsorbate molecules in the surface plane. The Langmuir model fits Cu (II) and Cd (II) adsorption isotherms in the concentration range *C*_eq_ = 0–10 mmol/L (Table 1). The essential characteristic of Langmuir isotherm can be expressed by the *R_L_* parameter, which defines the favorable conditions for an isotherm. If *R_L_* <1, the process is favorable, if *R_L_* = 1 the process is linear, and *R_L_* > 1 means the process is unfavorable [35]. As seen in Table 1, the adsorption process of Cd (II) and Cu (II) on the Fe_3_O_4_/SiO_2_-NH_2_-DTPA nanocomposite surface was favorable. According to the model, in an equilibrium concentration range *C*_eq_ = 0–10 mmol/L, the maximal adsorption capacity of Cd (II) and Cu (II) ions on Fe_3_O_4_/SiO_2_-NH_2_-DTPA nanocomposite was 0.30 and 0.07 mmol/g, respectively. 

The linearization for Freundlich equation was empirical, and applicable to adsorption on heterogeneous surfaces as well as multilayer adsorption. In our case, *R* values indicated quite poor correlation with the model, *R*^2^ = 0. 75380 for Cd (II) and *R*^2^ = 0.67160 for Cu (II).

### 2.3. Urease Immobilization and Activity in Presence of Cu (II) and Cd (II)

Urease immobilization on the surface of Fe_3_O_4_/SiO_2_-NH_2_-DTPA nanocomposite was conducted according to the method described in “Materials and Methods” section. Adsorption of urease is quite a rapid process, and its adsorption isotherm corresponds well to the Langmuir adsorption model. 

The samples of immobilized urease were then applied for the hydrolysis of pathologic concentrations of urea (20 mmol/L) in an isotonic buffer solution. The produced nanocomposites were tested for the stability of their enzymatic activity in a sequence of tests, where the material was time by time extracted magnetically, and then set in contact with a new portion of urea solution. 

**Figure 5:**
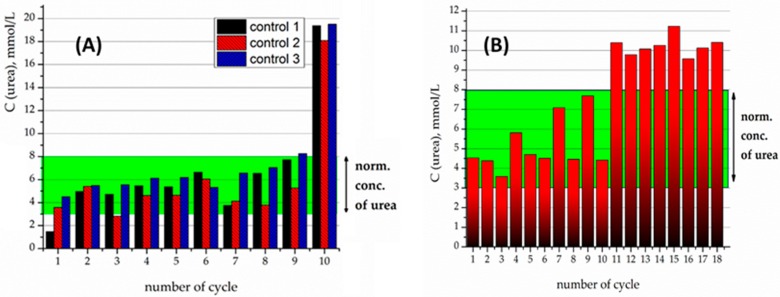
Hydrolysis of pathologic concentration of urea in the isotonic buffer by: (**A**) Fe_3_O_4_/SiO_2_-NH_2_-DTPA-Urease nanocomposite, 5% crosslinking by glutaraldehyde; three control iterations (cycle 10—blank, 20 mmol/L of urea); (**B**) Fe_3_O_4_/SiO_2_-NH_2_-DTPA-Urease nanocomposite, 5% crosslinking by glutaraldehyde; 18 cycles (contact time: cycle 1–10—2 min; cycle 11–18—1 min).

**Figure 6:**
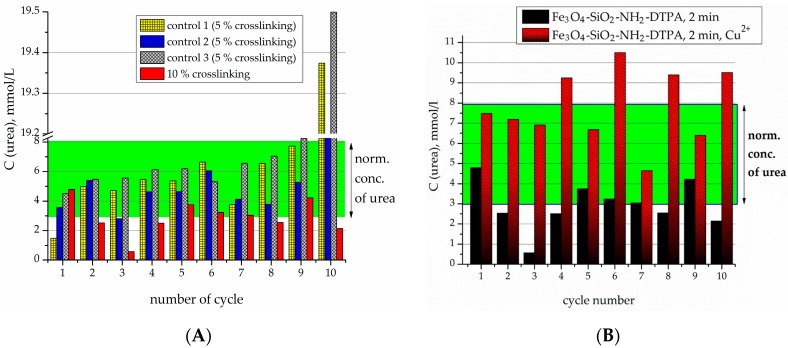
Hydrolysis of pathologic concentration of urea in the isotonic buffer by: (**A**) Fe_3_O_4_/SiO_2_-NH_2_-DTPA-Urease nanocomposite, 5, 10% crosslinking by glutaraldehyde; contact time: 2 min; (**B**) Fe_3_O_4_/SiO_2_-NH_2_-DTPA-Urease nanocomposite, 10% crosslinking by glutaraldehyde; *C* (Cu^2+^) = cycle number × 0.1 µmol; contact time: 2 min.

Crosslinking of urease by glutaraldehyde (5% and 10 wt %) on the surface of Fe_3_O_4_/SiO_2_-NH_2_-DTPA nanocomposite was performed in order to prevent urease desorption during repeated cycles of urea decomposition. The comparison of the observed residual concentrations of urea in the first 10 tests for each type of sample is made in Figure 5 and Figure 6. 

For the 5% crosslinked urease on the Fe_3_O_4_/SiO_2_-NH_2_-DTPA particles, urease activity persisted for 10 cycles, but tended to proceed beyond of a physiological level of urea (Figure 5A). For instance, on the 11th cycle of urea decomposition, its concentration decreased twice, but was higher than the normal level (Figure 5B). Urease activity persisted for 18 cycles.

The samples produced by 10% crosslinking of urease revealed an even higher stability and activity than 5% crosslinked ones (Figure 6A). For the particles with 10% crosslinking of urease, in all of the first 10 experiments, it was capable of decreasing the urea concentration to below the normal level. It can clearly be seen that the best effect in relation to stability and efficacy was demonstrated by the sample with 10% crosslinking.

For further experiments with heavy metals, the sample with 10% crosslinking was used. Previously it was shown that the presence of heavy metal cations tended to quench the activity of the urease [20,25]. Residual enzyme activity after the addition of heavy metals became concentration-dependent, clearly decreasing with an increase in heavy metal content in solution (0.1–1 µmol) [20,25]. Model cations Cd^2+^ and Cu^2+^ were chosen; these are typically known to rapidly and quantitatively decrease the enzyme activity via strong complexation [36,37].

It has been observed previously in the literature that the immobilized enzyme generally displays lower sensitivity to the environment, and in particular to the presence of heavy metal cations, compared to the native enzyme [38]. In case of the native enzyme, heavy metal cations are unhindered in interacting with the active center of the enzyme and can block it via complex formation [39].

In the micromolar range, in contrast to non-DTPA containing adsorbents, even when the concentration of Cu^2+^ reached 1 µmol/L (cycle 10), the Fe_3_O_4_/SiO_2_-NH_2_-DTPA-Urease composite was still capable of decreasing the urea concentration to normal levels—below 8 mmol/L (Figure 6B).

**Figure 7:**
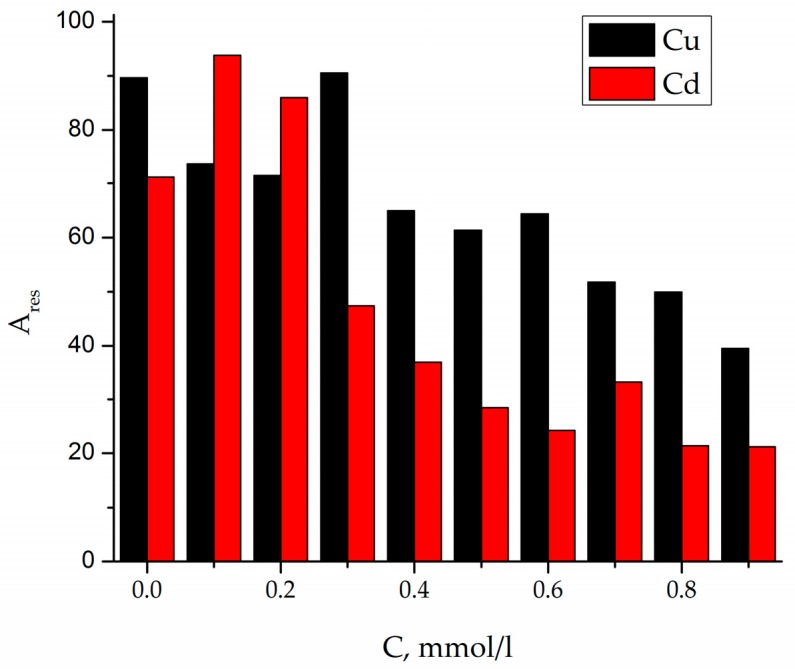
Residual activity of crosslinked urease on the surface of Fe_3_O_4_/SiO_2_-NH_2_-DTPA nanoadsorbents in presence of copper (II) and cadmium (II) ions.

For the Fe_3_O_4_/SiO_2_-NH_2_-DTPA-urease nanocomposite, the concentration-dependent decrease of activity in the presence of heavy metal ions was observed (Figure 7), but at higher metal concentrations, *C* (Cu^2+^ or Cd^2+^) = 0.1–1 mmol/L. For each cycle of urea addition, the concentration of Cu of Cd increased according to the equation: *C* (Me^2+^) = cycle number × 0.1 mmol/L. 

It could clearly be observed that immobilization of the enzyme on the surface of nanoparticles strongly improved its resistance to heavy metal poisoning. In the case of the Fe_3_O_4_/SiO_2_-NH_2_-DTPA composite, the immobilized enzyme was protected, on one hand, by the complexonate functions of DTPA groups of the matrix. They were conversely, able to contribute to the stabilization of the favorable configuration of the enzyme via chemical interactions with the functional groups that in some cases could be considered as mimicking the intracellular environment [40]. 

## 3. Discussion

The surface of the particles resembled that which has been generally observed for materials produced by the Stoeber synthesis. The surface was rather smooth and irregular, but similar to the surface of crystalline silica, with silanol groups pointed out into the solution. It is to these groups that the APTES is condensed in T^3^ or T^2^ fashion [41]. The distance between the grafted aminopropyl ligands was in the range 5.6–7.3 Å, predominantly 6.3 Å as can be deduced from the single crystal silsesquioxane models [42].

The amount of DTPA comprised only about a quarter of the amount of amino groups on the surface. Most probably, this is not because DTPA was bound by several amide functions (this would be difficult was there would be a long distance between the aminopropyl groups), but because not all grafting groups were available sterically (DTPA ligand need some space for motion). This was confirmed by a higher pH for the Pzc. In addition, aminopropyl radicals could be deactivated in relation to its ability to form an amide bond by its attraction to the silica surface via hydrogen bonds and/or electrostatic interactions in its protonated form dominating at neutral pH (R–NH_3_^+^∙∙∙∙∙SiO^−^). 

FTIR confirmed the presence of APTES and subsequent DTPA grafting to the nanocomposite surface. Presence of DTPA was demonstrated by peaks at 1636 and 1401 cm^−1^, corresponding to the symmetric (ν_s_) and asymmetric (ν_as_) stretching vibrations of the –COOH groups, respectively. A strong adsorption band (AB) at 1636 cm^−1^ indicates a non-coordinated –COO^-^ group. According to the literature data, -COOH group coordination on the composite surface (if any) can be characterized by four types of complexation: monodentate, bridging (bidentate), chelating (bidentate), and ionic interactions [43]. The type of carboxylate interaction with the composite surface can be distinguished by measuring wavenumber separation (Δ) between ν_as_ and ν_s_. If Δ smaller than 110 cm^−1^, binding is bidentate. If Δ is 200–320 cm^−1^, binding is monodentate. With regards to the types of complexation, we can conclude that DTPA on the surface was generating ionized –COO^−^ groups (see Table A2). 

An insight into the binding of metal cations to the adsorbent at higher solution concentrations could be obtained from the FTIR spectra presented on Figure A1a,b (Appendix A). Splitting of AB at 585 cm^−1^ (Fe_3_O_4_/SiO_2_-NH_2_- DTPA) to AB with two peaks at 580 and 619 cm^−1^ could be observed after metal adsorption (Fe_3_O_4_/SiO_2_-NH_2_- DTPA-Cu/Cd nanocomposites). AB at 619 cm^−1^ could be assigned to metal–O bonds (Table A1). The coordination geometries differed apparently for Cu and Cd, as the compositions of surface complexes differed. This is quite logical—Cd can have coordination numbers 4 (usually with larger donor atoms form Period 3 or below in the Periodic Table, such as S or Cl) or 6 (with smaller Period 2 donor atoms such as O or N), but quite regular. The Cu cation is Jahn-Teller distorted and can be in 4, 5 or 6-coordination even with smaller donor atoms. The bonding to Cu^2+^ cations is generally stronger than with Cd^2+^ ones. For the cadmium cations in this case, no difference in coordination geometry is expected between the inner sphere and outer sphere complexes (both hexa-coordinated), while for the copper cations, lower coordination numbers and stronger bonds are expected for the inner sphere complexes. For the outer spheres, the dominating form would be just the Cu(H_2_O)_6_^2+^ aqua complex with a weaker interaction with a carboxylate group of DTPA. This difference finds its reflection in the FTIR spectra of the adsorbents loaded with metal cations. The adsorption of Cd^2+^ resulted in some decrease in the intensity of the band at 1635 cm^−1^, reflecting the deprotonation of carboxylate groups and an asymmetric shift for the ν C–O band, where the maximum moved to 1411 cm^−1^. The appearance of the spectrum was not sensitive to the adsorbed amount of cadmium (Appendix A, Figure A1d). For the adsorption of copper cations, these effects were even more pronounced, with apparent splitting of the ν C–O band into two—at 1391 and at 1411 cm^−1^ (Appendix A, Figure A1c). The spectrum changed quite apparently with the increase in the amount of adsorbed copper, reflecting the changes in the geometry and bond strength for the carboxylate groups. This appeared quite symptomatic in the view of the differences in coordination between the two cations, as described above. 

The adsorption isotherms for both Cu^2+^ and Cd^2+^ cations are quite complex. Sorption processes on the functionalized surfaces can be characterized by inner- and outer-sphere complexation [44]. Outer-sphere mechanism involves electrostatic interaction, and depends on the ionic strength of the solution. During inner-sphere coordination (complexation), water molecules are substituted from the metal coordination sphere. According to that model, metal ions directly bind to functional (carboxy-) groups and do not “feel” the competition of background electrolyte ions. Typically, outer-sphere complexes, formed by electrostatic interaction, are less stable than inner-sphere surface complexes. It is worth noting that the composition of the first complex in copper adsorption equilibrium is quite peculiar with only 0.16 eq. (equivalents) of metal per DTPA ligand. This most probably indicates that the first step in the complexation involves not DTPA, but residual silanol groups known for their relatively high affinity to Cu^2+^ cations [45]. The relative stabilities of complexes formed by transition metals in aqueous solution are referred to as the Irving-Williams series. The place of metal ions in this series corresponds to the change in the second ionization potential, and their stability is due to the stabilization of complexes by the field of ligands: Cd^2+^ < Co^2+^ < Zn^2+^ < Ni^2+^< Cu^2+^. This means that the silanol groups of silica replace the water molecules in the internal coordination field of the aqua-metal ions as ligands that are capable of forming complex compounds on the surface [46]. An increase in the copper concentration results in formation of the complexes of Cu^2+^:DTPA ≈ 3:2 composition, indicating formation of chelate structure. According to Irving-Williams series, cadmium complexes with silanol groups have low stability and quickly hydrolyze on the surface. This fact can explain the relatively high *q*_e_ at *C*_eq_ = 10 mmol/L. The formation of chelated complexes with DTPA had apparently already begun at low Cd^2+^ concentrations. Further plateaus on adsorption isotherms for both metal cations could be connected with adsorption layer rearrangements. The final ratios of M^2+^:DTPA approached 4:1, being indicative of purely electrostatic surface interactions between cations and negatively charged –COO^−^ groups (four per grafted DTPA ligand). Modeling of the adsorption curves with equilibrium equations is meaningful when a single form of surface complex is stable in an appreciably broad interval of concentrations [47]. Therefore only the first adsorption step was evaluated in both cases. 

The strong bonding of metal cations with the introduced complexonate ligands apparently improves the stability of the immobilized enzyme, in which the functional active centers no longer are exposed to interaction with poisoning metal cations. Urease is efficiently immobilized due to cross-linking within a network on the surface, via coupling with glutaraldehyde. It conserves its activity in a long number of cycles, including washing and transfer of the particles, so that networking with glutaraldehyde seems to be a useful general approach for immobilization and grafting of enzymes on the surface of nanoparticles. A combination of complexonate and enzyme may open perspectives for adsorbents capable to maintain their enzymatic activity, and can potentially be regenerated after heavy metal adsorption, for example by eluation of cations in an acidic medium. 

Complexonate-grafted adsorbents may in perspective even be evaluated for the removal of uranium, thorium, and Rare Earth Elements (REE) in the view of affinity of these cations towards DTPA ligands and alike.

## 4. Materials and Methods 

The following reagents were used in the present study: tetraethoxysilane [TEOS, Si(OC_2_H_5_)_4_, Sigma-Aldrich, Cat. No. 8.00658 98%]; 3-aminopropyltriethoxysilane [APTES, (C_2_H_5_O)_3_Si(CH_2_)_3_NH_2_, Sigma-Aldrich, 99%]; iron(II) chloride tetrahydrate (FeCl_2_·4H_2_O, Sigma-Aldrich, Cat. No. 380024 97%); iron(III) chloride hexahydrate (FeCl_3_·6H_2_O, Sigma-Aldrich, Cat. No. 31232-M 98%); diethylene triamine pentaacetic dianhydride (DTPA anhydride, C_14_H_19_N_3_O_8_, Sigma-Aldrich, Cat. No. 284025 98%); water ammonia [NH_4_OH (25%)]; ethanol (96%); acetone (Sigma-Aldrich, 99.9%); K_2_HgI_4_ (Nessler’s reagent, Sigma-Aldrich, Cat. No. 345148); 0.1 M HCl; 0.1 M NaOH; 0.06 M phosphate buffer (pH 7.0); 0.1 M ethylenediaminetetraacetic acid (EDTA); urea powder (Sigma-Aldrich). Urease used in this work was derived from soybeans “Jack beans” [EC 3.5.1.5, activity 43 Un mg^−1^ (pH 7.0), Fluka]; CuSO_4_ anhydrous (Merck, Kenilworth, NJ, USA); CdSO_4_·8/3H_2_O (Riedel-de Haen AG, Sleeze, Germany). 

Magnetite was prepared by co-precipitation of iron(II) and iron(III) chlorides with ammonia under a nitrogen atmosphere [26]. The obtained Fe_3_O_4_ particles were spherical with an average diameter of about 12 nm, and a specific surface area of about 96 m^2^ g^−1^ [48]. 

Micrographs of the samples were obtained on a JSM 6060LA scanning electron microscope (Jeol, Japan) in the secondary electron mode at an accelerating voltage of 30 kV. The samples were mounted on a specimen stage coated with an adhesive. In order to prevent the buildup of surface charge and to obtain a contrast image, a thin continuous layer of gold was deposited onto the sample surface in vacuo by cathode sputtering. 

Contents of Si, S and Fe were measured using scanning electron microscopy combined with energy dispersive spectroscopy (SEM-EDS) with a Hitachi TM-1000-mDeX microscope (Department of Chemistry, Biocenter, SLU, Uppsala, Sweden). 

FTIR spectra of all samples were obtained with a Perkin Elmer FT-IR spectrometer Spectrum-100 (Beanconsfield, UK). A total of eight scans were carried out on wavenumbers from 400 cm^−1^ to 4000 cm^−1^, in transmittance mode. All spectra were smoothed and baseline corrected.

Thermo-gravimetric analysis was carried out in air at a heating rate of 10 °C min^−1^, using a Perkin-Elmer TGA-7 or a Pyris 1 device (Beanconsfield, UK).

Urease enzyme activity was determined by the rate of ammonia formation in the urea hydrolysis reaction at 25 °C [38]. In all cases, the average of three parallel experiments (the biggest difference between them being <10%) was used for activity determination. The error of measurement using the Student coefficient at a rugged probability of 0.95 was less than 10%.

*Adsorption studies*. Batch experiments were carried out in triplicate in a pH range from 2 to 6, a sorbent dose 0.015–0.025 g and an initial metal concentration 100 mg/dm^3^ in sodium acetate buffer (pH = 4.91). Batch tests of adsorption of metal ions were conducted by mixing 5 mL of proper aqueous phase containing CdSO_4_ or CuSO_4_ with 0.0025 g of Fe_3_O_4_/NH_2_-DTPA composite in a plastic tube and shaking mechanically using a laboratory shaker (VWR Digital Orbital Shaker 15, Radnor, OH, USA) for 120 min. After the pH of solution was stabilized and equilibrated, Fe_3_O_4_/NH_2_-DTPA composite was filtered and concentration of Cd (II) and Cu (II) was measured.

Adsorption, on the surface of nanocomposite was calculated by the formula: *q*_e_ = (*C*_0_ − *C*_eq_)V/m, where *C* and *C*_eq_—initial and equilibrium concentration of ions in solution, mg/ml; *V*—volume of solution (cm^3^); *m*—mass of adsorbent (g).

The pH of the point of zero charge pH_zpc_ was measured using the pH drift method [31,32]. The pH of the sorbent in the 0.01 M NaCl solution was adjusted to between 2 and 12 by adding 0.01 M NaOH and 0.01 M HCl. 0.02 g of the adsorbent was added to 5 cm^3^ of the solution and after 24 h the final pH was measured. 

### 4.1. Preparation of Magnetite Nanoparticles with a Monofunctional Surface Layer (3-Aminopropylpropyl Groups—Sample 1) 

A 250 mg (1 mmol) batch of Fe_3_O_4_ was treated with ultrasound for 10 min in 50 cm^3^ of water. Thereafter, pre-hydrolyzed alkoxysilanes were added to the suspension. Their prehydrolysis was carried out by mixing 5.0 cm^3^ of ethanol, 0.97 cm^3^ of APTES (4.1 mmol), and 1.0 cm^3^ of NH_4_F (1% solution in water). The resulting suspension was then stirred for 5 min. This solution was added to a suspension of magnetite. A total of 5.6 cm^3^ TEOS (25 mmol) was then added to achieve the ratio of reactants in aliquots (0.5 cm^3^ every 5 min) for one hour, and the suspension was then stirred for another 5 h (Figure 1A). The molar ratio of the components was Fe_3_O_4_/TEOS/APTES = 0.001/0.025/0.0041. The precipitate of modified magnetite was collected by a magnet, and washed with water (5 × 200 cm^3^), ethanol (2 × 100 cm^3^) and acetone (2 × 100 cm^3^). 

### 4.2. Modification of Sample 1 by DTPA Groups (Sample 2)

280 mg of Fe_3_O_4_/SiO_2_-NH_2_ composite were suspended in 20 cm^3^ of toluene and sonicated for 10 min. After sonication, 350 mg of DTPA anhydride in 20 cm^3^ of toluene was added to Fe_3_O_4_/SiO_2_-NH_2_ composite. 0.25 cm^3^ of glacial acetic acid was added in order to catalyze the reaction. The suspension was stirred for 6 h under N_2_ atmosphere at 80 °C. 

The obtained Fe_3_O_4_/SiO_2_-NH_2_-DTPA (Figure 1D,E) composite was separated by magnets and washed by toluene (2 × 50 cm^3^), ethanol (2 × 100 cm^3^), and acetone (2 × 100 cm^3^).

### 4.3. Urease Sorption Studies

To study the kinetics of urease adsorption, a batch of functionalized magnetite (0.01 g) was shaken in a test tube with 5 cm^3^ of urease buffer solution at a concentration of 6 g L^−1^. After 5, 10, 20, 30, 60 and 90 min, the solution above the precipitate was analyzed for enzyme content. In all cases, the amount of bound enzyme was evaluated by the difference between the urease used for immobilization and its residual content in solution. The latter was determined in the solution after adsorption, and in the washing water, from the enzyme activity compared to the specific activity of the enzyme. It was assumed that the specific activity of the enzyme remained the same as that of the native one. To build urease adsorption isotherms, buffer solutions with concentrations from 2.0 to 10.0 g L^−1^ were used. The process was carried out for 4 h at room temperature with periodical stirring.

Urease sorption was performed by a static method for 4 h at room temperature, applying periodical stirring. A batch of functionalized magnetite (0.02 g) was put into 5 cm^3^ of mixed phosphate buffer (pH 7.0) and EDTA solution (volume ratio of 9:1) containing 5 mg urease. The precipitate was then separated from the solution by a magnet and washed 5 times with 5 cm^3^ phosphate buffer. The overall adsorption of urease was determined by the difference between the urease used for immobilization and its residual content in solution.

### 4.4. Urease Immobilization

50 mg of urease in 10 cm^3^ of mixed phosphate buffer (pH 7.0) and EDTA solution (volume ratio of 9:1) was incubated with 200 mg of Fe_3_O_4_/SiO_2_-NH_2_-DTPA composite for 18 h. The sample was separated by magnets and washed acetone (2 × 5 cm^3^). The sample was gently dried under vacuum and was stored under a nitrogen atmosphere at 4 °C.

### 4.5. Crosslinking of Urease by Glutaraldehyde

250 mg of Fe_3_O_4_/SiO_2_-NH_2_-DTPA-Urease composite was cross-linked by 2 cm^3^ glutaraldehyde solution in 9:1 solution (0.5 mg/cm^3^ or 1.0 mg/cm^3^; 5 and 10% crosslinking, respectively) for two hours at 45 °C. The sample was washed with phosphate buffer and acetone, and was stored under a nitrogen atmosphere at 4 °C.

### 4.6. Urease Enzyme Activity

Urease enzyme activity was determined by the rate of ammonia formation in the urea hydrolysis reaction at 25 °C [49]. In all cases, the error of the activity measurements was estimated using the Student coefficient (*n* = 3) at a rugged probability of 0.95, was less than 10%. The samples of immobilized urease were then applied for hydrolysis of pathologic concentrations of urea (20 mmol/L) in an isotonic buffer solution. Each sample of the immobilized enzyme was reacted with 10 mL of the urea solution mimicking the blood plasma. 10 aliquots of the test solution at 10 mL each were sequentially kept for 10 min at 37 °C in a reactor containing a sample of immobilized urease. The residual content of urease was then determined using a test with the Nessler’s reagent (0.1 mL of urea solution being added to a mixture of 1 mL of Nessler’s reagent and 9.95 mL water). 

## 5. Conclusions

Sol-gel technology opens attractive general approaches to hybrid core-shell materials with a combination of different functional characteristics. Covering magnetic nanoparticles by silica protects their cores from the aggressive environments and facilitates the grafting of functional ligands. Condensation of APTES onto a silica surface opens up further potential functionalization via the formation of amide bonds. Only about one-fourth of the grafted amino groups can be transformed into amides because of such factors as steric hindrance, and also the bonding of protonated amino functions to silanol groups via hydrogen- and electrostatic bonds (as indicated by Pzc measurements). Grafted DTPA groups display strong activity in adsorbing cadmium and copper cations, with the mechanisms involving strong bonding of smaller amounts of heavy metals in supposedly chelated inner sphere complexes at their lower concentrations, and somewhat weaker in outer sphere complexes at higher concentrations. 

It can be concluded that cross-linking of immobilized urease by glutaraldehyde prevents its desorption and increases the number of urea decomposition cycles (at least 18 cycles with two-fold urea concentration decrease). Crosslinking of 10 wt % urease allows the effective decrease of urea concentration in 20 mmol/L model solutions to 2 mmol/L for up to 10 decomposition cycles. Due to presence of DTPA groups, Cu^2+^ ions in concentration 1 µmol/L do not significantly affect the urease activity.

It can clearly be seen that the best effect in relation to stability is demonstrated by the samples of urease immobilized on the particles with 10% crosslinking. In all the first 10 experiments, it was possible to decrease the urea concentration below the normal level. It could clearly be observed that immobilization of the enzyme on the surface of nanoparticles strongly improved its resistance to heavy metal poisoning.

## Figures and Tables

**Table 1:** Langmuir parameters for Cu (II) and Cd (II) adsorption on the surface of the Fe_3_O_4_/SiO_2_-NH_2_-DTPA nanocomposite.

Equilibrium Concentration Range *C*_eq_ = 0–10 mmol/L	Langmuir Model				
	*q*_e_ exp, mmol/g	*q*_0_	*K*_L_	*R*_L_	*R*^2^
Cd (II)	0.28	0.30	1.010	0.019	0.9624
Cu (II)	0.065	0.07	3.271	0.006	0.9914

## Appendix A

**Table A1:** AB and assignments for the Fe_3_O_4_/NH_2_-SiO_2_-DTPA-Cu/Cd nanocomposites.

Assigments	ν, cm^−1^
Cu–O (δ OCO) Cd–O (δ OCO)	619
δ C–C	792
ν C–O (COOH)	1391
ν C–O (COOH)	1411
δ CH_2_	1449
δ CH_2_	1471
NH– (amide I)	1527
ν C=O (COOH)	1578
OH (H_2_O + COOH)	1632

There are three representative types of coordination of the carboxylate (–COO^−^) group to metal ion(s): unidentate, bidentate, and bridging [43]. A metal ion interacts equally with the two oxygen atoms of the –COO^−^ group in the bidentate form, whereas it interacts with only one of those oxygen atoms in the unidentate form. In the bridging form, a metal ion interacts with one of those oxygen atoms, and another metal ion interacts with the other oxygen atom. When one of these metal ions is replaced by a hydrogen atom of a water molecule, the system is in the pseudo bridging form.

## Appendix B

The thermogravimetric data presented at Figure A2.

**Table A2:** Shifts for carboxylate AB for the Fe_3_O_4_/SiO_2_-NH_2_- DTPA-Cu/Cd nanocomposites.

Δ_as-s COOH_	DTPA:Metal (Ratio)
106 (bidentate)	DTPA:Cu 1:2.775
127	DTPA:Cu 1:0.65
139	DTPA:Cu 1:0.1625
177	DTPA:Cd 1:4
183	DTPA (non-mobilized)
